# Case report: Cerebral amyloid angiopathy-related inflammation in a patient with granulomatosis with polyangiitis

**DOI:** 10.3389/fneur.2023.1277843

**Published:** 2023-11-09

**Authors:** Rebecca M. Seifert, Michael Rauch, Randolf Klingebiel, Lennart-Maximilian Boese, Isabell Greeve, Martin Rudwaleit, Wolf-Rüdiger Schäbitz

**Affiliations:** ^1^Universitätsklinik für Neurologie, Evangelisches Klinikum Bethel, Bielefeld, Germany; ^2^Institut für diagnostische und interventionelle Neuroradiologie, Evangelisches Klinikum Bethel, Bielefeld, Germany; ^3^Universitätsklinik für Innere Medizin und Rheumatologie, Klinikum Bielefeld Rosenhöhe, Bielefeld, Germany

**Keywords:** case report, neuroinflammation amyloid, cerebral amyloid angiopathy-related inflammation, vasculitis, granulomatosis with polyangiitis, magnetic resonance imaging

## Abstract

**Background:**

Cerebral amyloid angiopathy-related inflammation (CAA-ri) defines a subacute autoimmune encephalopathy, which is presumably caused by increased CSF concentrations of anti-Aβ autoantibodies. This autoinflammatory reaction is temporally and regionally associated with microglial activation, inflammation and radiological presence of vasogenic edema. Clinical characteristics include progressive demential development as well as headache and epileptic seizures. In the absence of histopathologic confirmation, the criteria defined by Auriel et al. allow diagnosis of probable resp. possible CAA-ri. CAA-ri shows responsiveness to immunosuppressive therapies and a possible coexistence with other autoinflammatory diseases.

**Methods:**

We present a case report and literature review on the diagnosis of CAA-ri in a patient with known granulomatosis with polyangiitis (GPA).

**Results:**

Initially, the presented patient showed neuropsychiatric abnormalities and latent arm paresis. Due to slight increase in CSF cell count, an initial antiviral therapy was started. MR tomography showed a pronounced frontotemporal edema as well as cerebral microhemorrhages, leading to the diagnosis of CAA-ri. Subsequent high-dose steroid treatment followed by six intravenous cyclophosphamide pulses resulted in decreased CSF cell count and regression of cerebral MRI findings.

**Conclusion:**

The symptoms observed in the patient are consistent with previous case reports on CAA-ri. Due to previously known GPA, we considered a cerebral manifestation of this disease as a differential diagnosis. However, absence of pachymeningitis as well as granulomatous infiltrations on imaging made cerebral GPA less likely. An increased risk for Aβ-associated pathologies in systemic rheumatic diseases is discussed variously.

## 1. Introduction

Cerebral amyloid angiopathy-related inflammation (CAA-ri) defines a subacute autoimmune encephalopathy, which is presumably caused by increased CSF concentrations of anti-Aβ antibodies targeting vascular amyloid depositions within the walls of leptomeningeal arteries, arterioles and capillaries (1). This autoinflammatory reaction is temporary and regionally associated with microglial activation, neuroinflammation, and the appearance of vasogenic edema in MR tomography (1–4).

Despite its well-known pathophysiology, the causes of CAA-ri development remain comparatively unclear. To date, sparse evidence from observational studies suggests a potential connection between CAA-ri and the presence of autoinflammatory diseases (5–8). On a more generalized level, various studies have investigated the connection between systemic autoinflammation and development of an amyloid pathology. In this context, previous animal data suggest an increased risk for the development of AD in bacterially-mediated systemic inflammation (9). This relevance of chronic inflammatory processes in dementia development seemed to be further supported by observations demonstrating a remarkable cognitive deterioration in AD patients with high TNF-α levels (10, 11). To antagonize this effect, non-steroidal anti-inflammatory drugs (NSAID) were supposed to protect against demential development. However, a meta-analysis in 2015 demonstrated that this effect was not statistically significant (11, 12). In the following time, the influence of specific anti-inflammatory diseases on the development of amyloid pathologies was investigated in more detail. These studies have so far yielded variable results: Whereas a > 2-fold increase in dementia risk has been described in systemic lupus erythematosus (13), such a correlation could not be established with certainty for other rheumatologic diseases (13–15). A possible explanation for these diverging results might be the high heterogeneity of the investigated study collectives and the involvement of pro- as well as anti-inflammatory cytokines in the autoinflammatory processes (11).

In particular with respect to granulomatosis with polyangiitis (GPA), a small vessel vasculitis, a potential association with the development of an amyloid pathology remains unclear to date. This fact might – at least in part – be explained by the low overall prevalence of CAA-ri (1, 16). In the following case report, we describe a patient diagnosed with CAA-ri in the concomitant presence of GPA using clinical and radiological criteria (17). Additionally, we review current evidence on the development of amyloid pathologies in patients with systemic rheumatic disease.

## 2. Case description

### 2.1. Clinical description

A 61-year-old patient presented at the A&E department in November 2022 after an out-of-hospital MRI had revealed abnormal findings with a suspected diagnosis of brain tumor or encephalitis.

Anamnestically, colleagues had described several months of a profound character change associated with difficulties in word finding and concentration. The patient had a history of osteoporosis, a depressive episode until 06/2021 (last treated with sertraline), a phase of malnutrition 11/2019 and a both histologically as well as chemically proven granulomatosis with polyangiitis (GPA).

At presentation, the clinical neurological examination revealed an alert, fully oriented patient without cranial nerve disorders, ataxia or sensory deficits. The reflex status was unremarkable without pyramid tract signs, and the patient did not show any gait or standing abnormalities. However, there was latent paresis with slight pronation of the right arm. Additionally, the patient showed severe neuropsychiatric abnormalities with word finding and comprehensive disorders, which also impeded the establishment of focused anamnesis.

**Figure 1:**
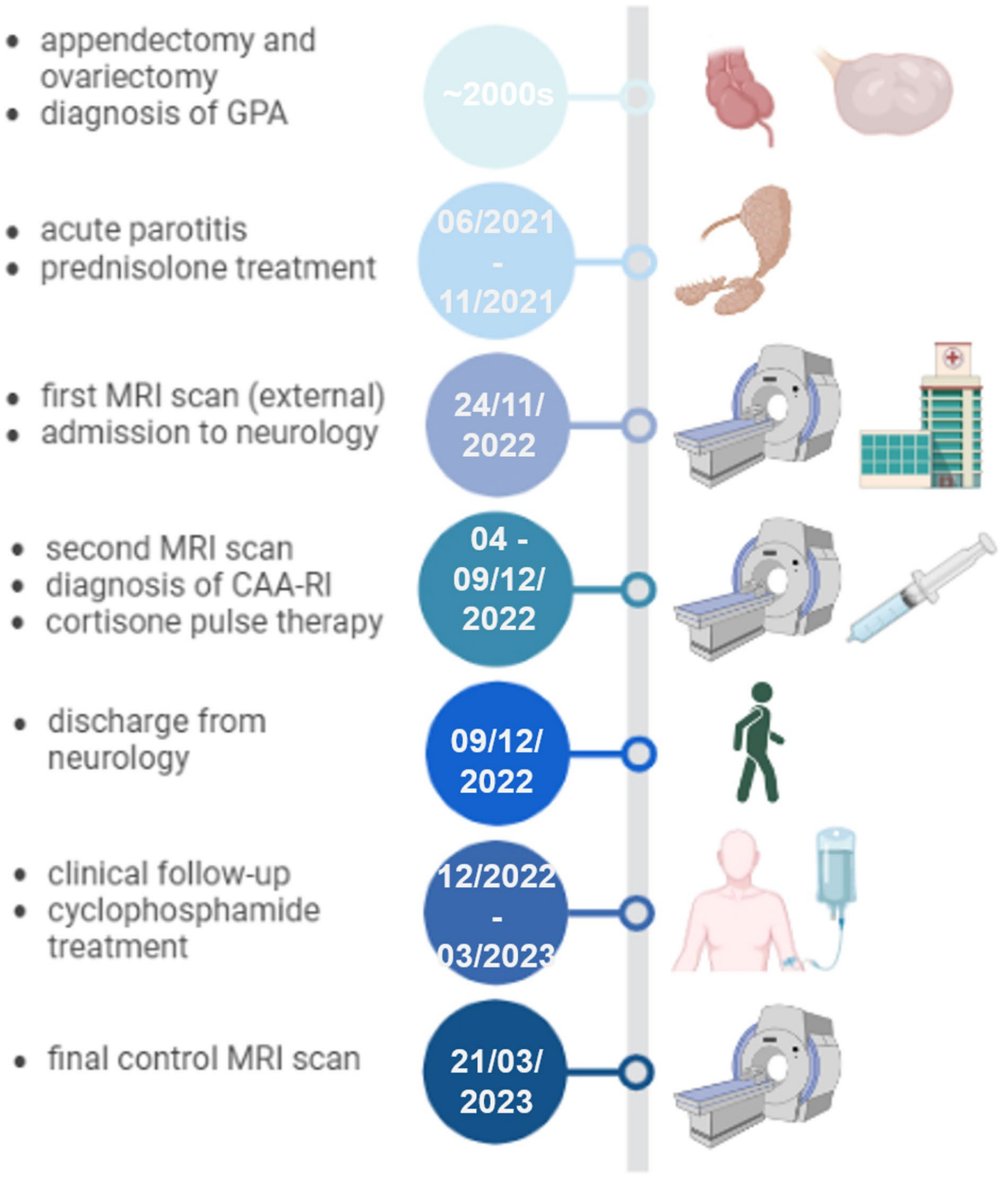
Timeline of the clinical presentation, relevant diagnostic and therapeutic interventions. CAA-ri: cerebral amyloid angiopathy-related inflammation, GPA: granulomatosis with polyangiitis, MRI: magnetic resonance imaging. Figure created in BioRender.com.

The patient was admitted for inpatient treatment including lumbar puncture as well as repetition of MR tomography (Figure 1). Based on clinical and radiological findings, we suspected CAA-ri using the diagnostic criteria established by Auriel et al. (17). Therefore, intravenous steroid treatment with 500 mg methylprednisolone for a total of five days (cumulatively 2,500 mg) was initiated. During this period, neuropsychological testing showed slight impairments in the areas of naming, word finding and word fluency without visuoconstructive or sensorimotor deficits. The latent arm paresis and initial word finding disorders gradually improved under steroid treatment.

Preceding discharge in 12/2022, a second CSF analysis as well as follow-up MRI scan were performed. Clinically, there was regression of neuropsychiatric changes except for a mild naming disorder. At discharge, prednisolone treatment was tapered from 80 mg p.o. daily to 60, 40, and 20 mg at two-week intervals of dose reduction.

To ensure long-term stabilization of the newly diagnosed probable CAA-ri, we conducted a neurological as well as rheumatological follow-up from 12/2022 to 04/2023. This included addition of six intravenous cyclophosphamide pulses, as well as final MRI control on 21/03/2023. Within this episode, normalization of neurological symptoms as well as an almost complete regression of cerebral lesions could be observed.

### 2.2. Laboratory results

In the present case, the diagnosis of probable CAA-ri was established using clinical and radiological criteria. At admission (24/11/2022), the initial lumbar puncture showed a leukocyte count of 11/μL with negative oligoclonal bands, a protein concentration of 563 mg/L (reference value: < 450 mg/L) and no cytological abnormalities. Based on these results, we initially suspected a viral meningoencephalitis, and started a calculated 11-day virostatic therapy with acyclovir. Subsequent tests for HSV-1/2, VZV, borrelia and syphilis were all negative. During the course of initial treatment, a second CSF analysis preceding discharge in 12/2022 revealed a declining cell count of 6/μL and no detection of antineural antibodies (Supplementary Table 1), thus indicating responsiveness to immunosuppressive therapy.

### 2.3. Imaging results

**Figure 2:**
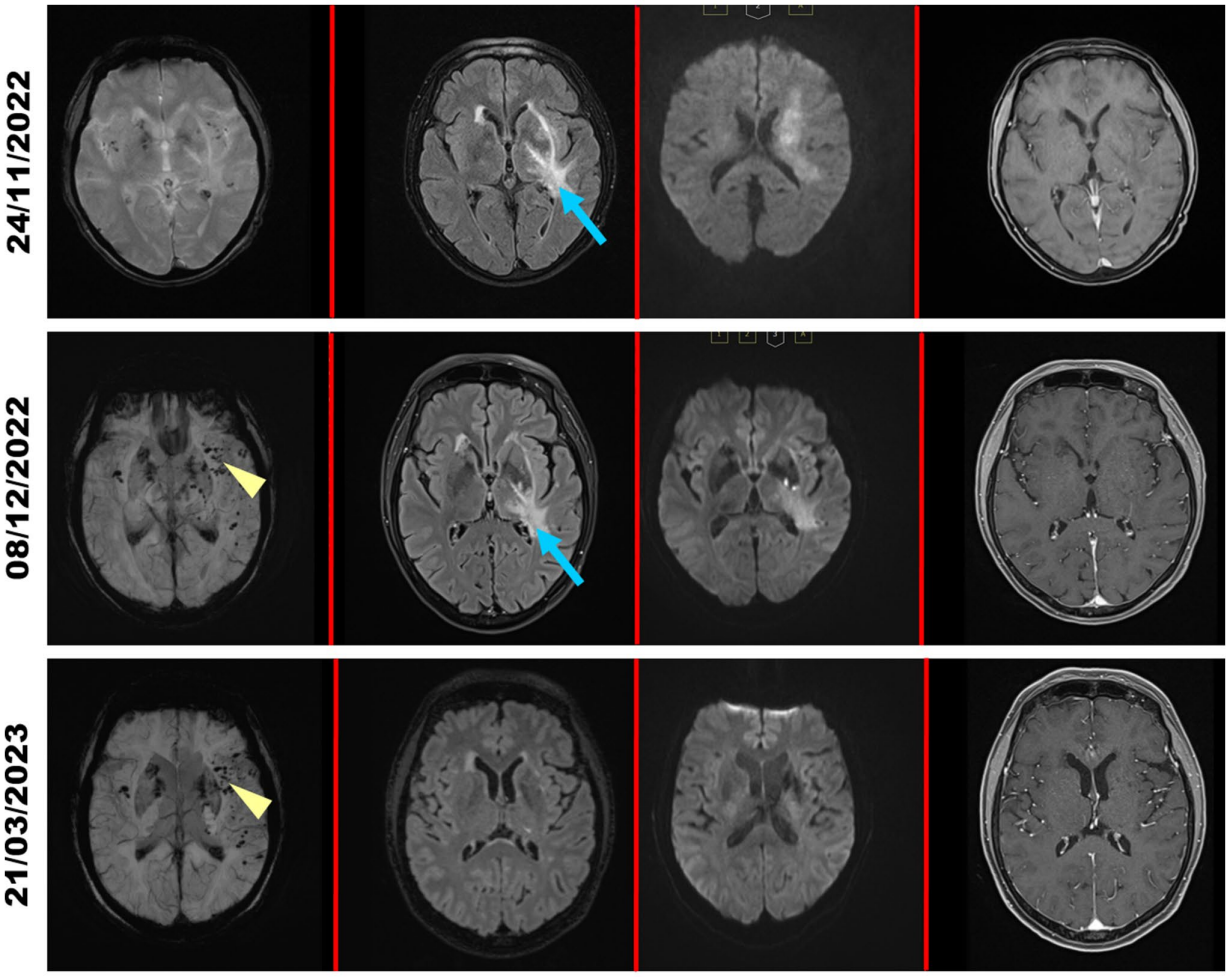
MRI scans during the clinical course. MRI scans of the patient at admission (24/11/2022; upper row), during corticosteroid treatment (08/12/2022; middle row) and in clinical follow-up in March 2023 (21/03/2023; lower row). Images were acquired (from left to right) in the SWI, FLAIR, DWI (*b* value = 1,000 s/mm^2^) and contrast-enhanced T1 sequence. At admission, the left frontotemporal region shows a pronounced frontotemporal edema (blue arrows) with significant reduction under corticosteroid treatment and complete regression in the clinical follow-up 03/2023. The SWI sequence shows microhemorrhages (yellow arrowheads) as a sign of newly diagnosed CAA-ri.

MR tomography showed a pronounced frontotemporal edema with involvement of the basal ganglia as well as cerebral microhemorrhages, so that we suspected probable CAA-ri based on the clinicoradiological diagnostic criteria established by Auriel et al. (17) (Figure 2, upper row). Corresponding to the CSF changes and clinical improvement, an MRI control scan preceding discharge in 12/2022 showed mild regression of the frontotemporal edema and perivascular contrast enhancement (Figure 2, middle row). Subsequent follow-up after discontinuation of cyclophosphamide treatment in 03/2023 demonstrated an almost complete regression of cerebral lesions (Figure 2, lower row).

### 2.4. Rheumatological workup

Difficult for the establishment of the correct diagnosis was the simultaneous presence of granulomatosis with polyangiitis (GPA) in the patient here described. Around the 2000s, the patient had undergone appendectomy and ovariectomy due to ruptured mucinous cystic neoplasia in the mesentery (Figure 1). Of note, histology of surgical specimen obtained at ovariectomy revealed adnexitis highly suggestive of granulomatosis with polyangiitis. At this point of time, proteinase-3 antibodies and c-ANCA were slightly elevated but there was no other organ involvement of GPA and, therefore, no need for treatment. The patient was followed up regularly without any immunosuppressive treatment until 06/2021. Herein, a single episode of acute parotitis occurred, which responded well to a short prednisolone treatment after antibiotic failure. Subsequently, the clinical course had been unremarkable again without clinical GPA manifestations and normal C-reactive protein, but constantly elevated anti-proteinase-3 antibodies (92-100 IU/mL, normal <2.0–3.0 IU/mL) and elevated c-ANCA 1:1280 (normal <1:20 only), so that no further immunosuppression had been initiated until the time of neurological admission in 11/2022.

Due to this rheumatological history, a cerebral manifestation of GPA could not be excluded with certainty, although it was much less likely because of the radiological absence of typical granulomas or pachymeningopathy (18). For therapeutic monitoring of GPA, we tested the c-ANCA and proteinase-3 autoantibody levels before and after completion of immunosuppressive treatment. Upon this therapy, anti-proteinase-3 levels decreased from 107 IU/mL (12/2022) to 28 IU/mL (04/2023) and c-ANCA from 1:1280 to 1:150. Of note, while CRP stayed normal throughout the entire disease course, serum amyloid A (SAA) was markedly elevated (84.5 mg/L, normal <6,4 mg/L) before initiation of cyclophosphamide treatment. Increased levels of SAA have been described in patients with various rheumatologic diseases, including GPA (19). Herein, high SAA levels correlate with increased disease activity, so that SAA might be used as a marker for therapeutic monitoring (20). After completion of cyclophosphamide treatment, SAA levels dropped to 12.1 mg/L in 04/2023 accompanying the therapeutic success in accordance with the underlying diagnosis of GPA.

## 3. Discussion

CAA-ri shows a variety of clinical manifestations, hence occasionally complicating differential diagnosis to other acute neurological, inflammatory, neurodegenerative or malignant diseases. In patients with diagnosed AD, concomitant presence of cerebral amyloid angiopathy (CAA) could be observed in more than 80% of described cases (21). Additionally, a subset of AD patients – either under treatment with amyloid-clearing therapies or spontaneously – showed histological and radiological signs of CAA-ri as well as elevated anti-Aβ autoantibodies (3, 22–25). These findings indicate clinical overlap between different amyloid-associated diseases. Moreover, differential diagnosis to low-grade gliomas may be complicated by the presence of extensive brain edema, as seen in the patient here described (Figure 2, first row) (26, 27). Such a “tumefactive” presentation of CAA-ri is more frequently observed in elderly patients due to white matter lesions surrounded by perifocal edema (27). As described by Ronsin and colleagues, misdiagnosis of CAA-ri as low-grade brain tumor occurred more frequently if initial MR imaging was conducted without T2* or SWI sequences for identification of cortical or subcortical microbleeds (27). In line with this data, we performed initial SWI imaging in the patient here described, which confirmed the underlying diagnosis of CAA-ri due to the presence of multiple cerebral microbleeds (Figure 2, first row).

Common clinical leading symptoms of CAA-ri include progressive dementia with disturbances of perception, memory and word finding; however, psychopathological abnormalities with paranoid-delusional experiences, seizures, headaches or sensory disturbances may also occur (1, 28, 29). These principal symptoms were also present in the patient here described in the sense of the observed personality change with memory as well as word finding disorders. Headache or epileptic seizures did not occur at any time in the presented case report.

Joint occurrence of CAA-ri with other autoinflammatory diseases (here described: GPA) generally raises the question whether there might be an association between both disease entities. Literature research regarding similar cases indicates a coexistence of CAA-ri with autoimmune thyroid disorders (hypothyroidism or Grave’s disease) (5), as well as previously known rheumatoid arthritis (5–7). Though less frequently, common presence with sarcoidosis (30), autoimmune hepatitis (5) or psoriasis (31) have also been described. Additionally, Fouret et al. could recently establish the diagnosis of CAA-ri in a patient with known giant cell arteriitis (8). However, to date there is no data on a coexistence of CAA-ri with other vasculitis, especially GPA. According to Danve et al., the observed joint occurrence of CAA-ri with other autoinflammatory diseases might be explained by autoimmunity as a unifying pathomechanism of both conditions (5). Many of the observed CAA-ri cases in patients with autoinflammatory diseases have been confirmed via brain biopsy (5–8), as MR tomography showed a mass lesion with extensive perilesional edema (2). These MR tomographic changes were also described as adverse effects of amyloid-modifying therapies in a subset of AD patients (22–24), in which determination of anti-Aβ autoantibodies might allow risk stratification preceding therapeutic intervention (2, 4, 25, 28, 32). Regarding the described joint occurrence of CAA-ri and giant cell arteritis, biopsies of both the cerebral lesion as well as the superficial temporal artery were carried out to confirm both diagnoses (8). In the patient here described, we forewent a brain biopsy due to positivity of the diagnostic criteria for probable CAA-ri (17) and the observed remarkable clinical as well as radiological improvement under immunosuppressive therapy (Figure 2).

Difficult for the diagnostic workup was the concomitant presence of GPA in the present case report. Cerebral involvement is also possible in the setting of GPA, although this is a relatively rare manifestation, accounting for approximately 7–11% of described cases (18). To date, there is no common pathognomonic feature of central nervous system GPA. However, the observed MR tomographic changes allow classification of two forms of progression: The granulomatous type (G-CNS) is characterized by focal or diffuse meningeal thickening due to hypertrophic pachymeningitis. This inflammatory process might also include the spinal cord or pituitary gland, which then appears enlarged with homogenous or heterogenous contrast enhancement and focal thickening of the infundibulum (18, 33). de Luna et al. also defined a vasculitic form of progression (V-CNS), in which ischemic or hemorrhagic strokes as well as white matter lesions could be observed. The latter occur due to vasculitic processes and typically show a vascular distribution (18, 33). In contrast to differential diagnoses, central nervous system GPA does not show mass lesions in imaging (33). Brain imaging criteria are the most reliable and established features to distinguish between CAA-ri and central nervous system GPA (summarized in Table 1). According to these, we suspected probable CAA-ri in the patient here described (Table 1, left column) (1, 2, 17), whereas a GPA with cerebral involvement seemed much less likely based on the imaging criteria (Table 1, right column).

**Table 1:** Radiological comparison of CAA-ri and central nervous system GPA. Based on the criteria by Auriel et al. (17), the diagnosis of CAA-ri can be established by consideration of four main diagnostic aspects (left column). In contrast, central nervous system GPA shows a granulomatous or vasculitic form of progression, occasionally combined with systemic GPA symptoms (right column).

CAA-RI	Central nervous system GPA
1. White matter lesions (T2-FLAIR) a) Asymmetric white matter hyperintensities b) Symmetric white matter lesions reaching the immediately subcortical white matter c) Involvement of cortex may be seen and predispose to seizures 2. Hemorrhagic lesions (T2*/ SWI) a) Cerebral macrobleed (macrohemorrhage) b) Cerebral microbleed (microhemorrhage) c) Cortical superficial siderosis 3. Leptomeningeal/ vascular involvement a) Leptomeningeal contrast enhancement in approximately half of patients 4. MR angiography a) May show medium-sized arteries involved b) Multisegmental stenosis possible c) Wall thickening and enhancement (not specific)	1. No pathognomonic features 2. Possible features: a) Intracerebral or meningeal granulomatous lesions (hypertrophic pachymeningitis) b) Small vessel CNS vasculitis leading to infarctions c) Intracranial hemorrhage d) Chronic systemic arteritis involving lungs, kidney and sinus may help in differentiating

Research of past years has shown that autoinflammatory processes play a crucial role in the pathogenesis of Aβ-associated diseases. Thus, significantly elevated levels of anti-Aβ-40 and Aβ-42 have been detected in patients with active CAA-ri compared to healthy controls (32). An autoinflammatory component of such diseases is further supported by suggestive evidence demonstrating that CAA-ri responds to immunosuppressive therapies (28, 29). According to data from Antolini and colleagues, sufficient control of inflammatory processes might also have a major prognostic effect, as patient outcome seems to be independent from radiological abnormalities, or mutations in apolipoprotein metabolism (1). Additionally, clinical improvement or disease stabilization could more likely be achieved by initiation of immunosuppression (29). As shown in a case–control study by Regenhardt and colleagues, sole corticosteroid treatment leads to clinical improvement in CAA-ri patients compared to a non-treated cohort (97% vs. 50% improvement, OR 26.0) (29). In this context, slow oral tapering is recommended to prevent the development of recurrences (HR 4.68, *p* < 0.006) (1). However, individual therapeutic strategies with azathioprine (1), cyclophosphamide (1, 29), or mycophenolate mofetil (MMF) (29), have already been described. In accordance with this data, we present a case where a cortisone pulse therapy with slow oral tapering followed by cyclophosphamide treatment was selected due to the concomitant diagnosis of GPA.

Although a coexistence of CAA-ri with autoimmune diseases has currently been observed in various cases (5–8, 30, 31), a causal relationship between CAA-ri development and GPA is not certain at this time. The observed common occurrence of CAA-ri with giant cell arteritis, as well as current descriptions of white matter lesions in patients with subclinical cognitive impairment due to small vessel vasculitis (34), support the idea of a pathophysiological connection between both disease entities.

## 4. Conclusion

- In the present case report, clinical and radiological criteria were used for the diagnosis of CAA-ri in a patient with known GPA.
- Multiple clinical manifestations are possible in the setting of CAA-ri, the most common being progressive dementia, headache and epileptic seizures.
- A connection between systemic autoinflammatory diseases and an increased risk for developing amyloid pathologies is currently matter of debate.

## Data availability statement

The original contributions presented in the study are included in the article/Supplementary material, further inquiries can be directed to the corresponding author.

## Ethics statement

Ethical approval was not required for the studies involving humans in accordance with the local legislation and institutional requirements. The participants provided their written informed consent to participate in this study. Written informed consent was obtained from the individual(s) for the publication of any potentially identifiable images or data included in this article.

## Author contributions

RS: Conceptualization, Formal analysis, Investigation, Methodology, Visualization, Writing – original draft. MR: Conceptualization, Formal analysis, Investigation, Supervision, Writing – original draft, Methodology. RK: Methodology, Writing – original draft, Data curation, Formal analysis, Resources. L-MB: Writing – original draft, Data curation, Formal analysis, Resources. IG: Writing – review & editing, Supervision. MR: Investigation, Formal analysis, Resources, Writing – review & editing. W-RS: Investigation, Project administration, Supervision, Writing – review & editing, Conceptualization, Resources.
