# Supplementary material for: Case report: Cerebral amyloid angiopathy-related inflammation in a patient with granulomatosis with polyangiitis
Source: Front Neurol. 2023 Nov 9;14:1277843. doi: 10.3389/fneur.2023.1277843 (PMC10666051; doi:10.3389/fneur.2023.1277843)
Supplement: Supplementary file 1 [file Table_1.DOCX]

Supplementary Material

# Supplementary Table 1

| Protein | Result (patient) | Dimension | Reference value |
| --- | --- | --- | --- |
| DPPX | <1:20 | Titer | <1:20 |
| LGI1 | <1:20 | Titer | <1:20 |
| CASPR2 | <1:20 | Titer | <1:20 |
| Glycine receptor | <1:20 | Titer | <1:20 |
| mGluR5 | <1:20 | Titer | <1:20 |
| GAD65 | <1:1 | Titer | <1:1 |
| NMDAR | <1:1 | Titer | <1:1 |
| GABAAR | <1:1 | Titer | <1:1 |
| GABABR | <1:1 | Titer | <1:1 |
| IgLON5 | <1:1 | Titer | <1:1 |
| AMPAR1/2 | <1:1 | Titer | <1:1 |
| DPPX | <1:1 | Titer | <1:1 |
| LGI1 | <1:1 | Titer | <1:1 |
| CASPR2 | <1:1 | Titer | <1:1 |
| Glycine Receptor (in CSF) | <1:1 | Titer | <1:1 |
| mGluR5 (in CSF) | <1:1 | Titer | <1:1 |
| mGluR1 (in CSF) | <1:1 | Titer | <1:1 |
| Neuronal Antibodies (Immunoblot-IgG) | | | |
| Amphiphysin | Negative |  | Negative |
| CV2/CRMP5 | Negative |  | Negative |
| Ma2/Ta (PNMA2) | Negative |  | Negative |
| Ri | Negative |  | Negative |
| Yo | Negative |  | Negative |
| Hu | Negative |  | Negative |
| Recoverin | Negative |  | Negative |
| Sox1 | Negative |  | Negative |
| Titin | Negative |  | Negative |
| Zic4 | Negative |  | Negative |
| DNER/Tr | Negative |  | Negative |
| Amphiphysin (in CSF) | Negative |  | Negative |
| CV2/CRMP5 (in CSF) | Negative |  | Negative |
| Ma2/Ta (PNMA2) (in CSF) | Negative |  | Negative |
| Ri (in CSF) | Negative |  | Negative |
| Yo (in CSF) | Negative |  | Negative |
| Hu (in CSF) | Negative |  | Negative |
| Recoverin (in CSF) | Negative |  | Negative |
| Sox1 (in CSF) | Negative |  | Negative |
| Titin (in CSF) | Negative |  | Negative |
| Zic4 (in CSF) | Negative |  | Negative |
| DNER/Tr (in CSF) | Negative |  | Negative |
| Tissue-based assay | | | |
| Anti-Neuropil | Negative |  | Negative |
| Anti-Purkinje cells | Negative |  | Negative |
| Anti-GFAP | Negative |  | Negative |
| Anti-adenylate kinase | Negative |  | Negative |
| Anti-ANNA3 | Negative |  | Negative |
| Anti-Neurexin-3α | Negative |  | negative |

**Supplementary Table 1:** Titers of antineuronal antibodies investigated in the patient.

During lumbar puncture preceding dismission in December 2022, a CSF serum pair of samples was acquired and investigated for the presence of antineuronal antibodies using a standard panel. Presence of antineuronal antibodies was tested via cell-based immunofluorescence, immunoblot and tissue-based assays.
